# Compatibility Optimal Design of Axially Loaded Circular Concrete-Filled Steel Tube Stub Columns

**DOI:** 10.3390/ma14174839

**Published:** 2021-08-26

**Authors:** Jinglin Fan, Fei Lyu, Faxing Ding, Dan Bu, Siqing Wang, Zhongkun Tan, Sui Tan

**Affiliations:** 1 School of Architecture and Art, Central South University, Changsha 410075, China; jinglin2021@csu.edu.cn; 2 School of Civil Engineering, Central South University, Changsha 410075, China; dinfaxin@csu.edu.cn; 3 Hunan Architectural Design Institute Group Co., Ltd., Changsha 410012, China; bonannee@126.com (D.B.); AG5678@163.com (S.W.); 4 Hunan Zhongteng Civil Engineering Technology Co., Ltd., Changsha 410075, China; tzk9888@hnu.edu.cn; 5 National Engineering Laboratory for High Speed Railway Construction, Changsha 410075, China; sunnytansui@csu.edu.cn

**Keywords:** concrete-filled steel tubular stub columns, axial load–strain curve, compatibility, optimal design

## Abstract

Numerous studies have been carried out on the axially loaded circular concrete-filled steel tube (CCFST) stub columns. However, to date, no clear evaluation criterion for the compatibility of its design parameters has been established. In the present study, the compatibility of the design parameters (concrete compressive strength $f_{c}$, steel yield strength $f_{y}$, diameter *D* and thickness of steel tube *t*) of axially loaded CCFST stub columns was quantitatively investigated in terms of the contribution of the composite actions to the axial bearing capacity of the columns. The composite ratio λ was proposed as an indicator to represent the effectiveness of the composite actions. A numerical framework of the determination of λ was established, making use of a series of existing widely recognized constitutive models of structural steel and concrete. Some modifications were carried out on these models to ensure the numerical stability of the presented analysis. Moreover, the rationality of the combined use of these models was verified. The analytical results show that excessive or very small D/t ratio should be avoided in design. Meanwhile, the combined use of low-strength steel and high-strength concrete should be avoided. A table of optimal D/t ratios corresponding to different material strength matches was provided for designers. Finally, an optimization of the design parameters using the proposed method and the existing design specification was performed.

## 1. Introduction

Over the past two decades, concrete-filled steel tube (CFST) columns have been experimentally and theoretically verified to have far superior strength, ductility, and energy absorption capacity compared to hollow thin-walled steel columns and reinforced concrete (RC) columns [1]. In view of their merits, the potential for the application of CFST columns in high-rise and large-scale buildings has long been recognized, and they have been applied in engineering practice in projects such as the Taipei 101 tower, Two Union Square in Seattle, WA, USA, the Shimizu High Rise Building in Tokyo [2], and more recently, KK100 in Shenzhen, China, and Alipay Headquarters Building in Hangzhou, China. Additionally, with the intense research focus on the prefabricated building and accelerated bridge construction in recent years, the application of CFST columns in low-to-moderate buildings and bridge piers [3,4] has been increasingly promoted due to the features such as the elimination of frameworks and factory prefabrication to enable easy construction. The benefits of CFST columns mainly accrue from the combined advantages of different constituent materials and the elimination of the shortcomings of steel and concrete; namely, the outer steel tube provides lateral confinement to the in-filled concrete, and the in-filled concrete prevents the inward local buckling of the steel tube [5]. Currently, several cross-section types of CFST columns are commonly exploited in the building construction, such as rectangular, circular, and octagonal. In particular, circular CFST (CCFST) columns can provide the greatest confinement effects [6] as well as an elegant structural form and thus have been preferred by structural engineers worldwide. Meanwhile, good impact resistance [7], stability [8] and corrosion resistances [9] of CFST columns were reported. Because of the above advantages, composite structural columns based on the similar concept, such as recycled aggregate concrete-filled steel tube [10], concrete-filled aluminum alloy tube [11], lightweight concrete-filled steel tube [12], stirrup-confined concrete-filled steel tube [13], and concrete-filled dual steel tube columns [14] were proposed in recent years to satisfy different design objectives for low carbon, good appearance, being lightweight and high strength, etc.

For support members, e.g., building columns and bridge piers, the axial compressive capacity is recognized as the most significant and fundamental structural characteristic of safe design. Therefore, the axial compressive behaviors and analytical prediction of axial compression capacity of CFST columns have been continuously investigated in CFST column research. In the 1970s, researchers including Furlong [15], Gardner and Jacobson [16], and Knowles and Park [17] had already investigated the axially loaded behaviors of CFST columns via laboratory testing and finite element modeling. It was found that the performance of axially loaded CFST columns is heavily affected by the diameter-to-thickness ratio (width-to-thickness ratio for rectangular columns) and height-to-diameter ratio (width-to-height ratio for rectangular columns). Following this insight, numerous researchers have contributed to the testing of CFST columns with different dimensions [18,19], material strength grades [20,21], and cross-section types [22,23] over the past three decades and have proposed empirical formulas [24] for calculating the axial compression capacity and effective limitations of design parameters such as the aspect ratio and slenderness-to-thickness ratio. Those suggestions were adopted by various design specifications including Eurocode4 [25], AISC [26], and GB50936 [27].

Numerous studies have been carried out on the axially loaded circular concrete-filled steel tube (CCFST) stub columns. However, to date, no clear evaluation criterion for the compatibility of its design parameters has been established. Although the mechanical behaviors of axially CCFST stub columns have been extensively investigated, and the scopes of geometric and physical parameters were presented in the specifications [1], the guidance regarding the compatibility of design parameters have been only qualitative. For example, in the latest specification of T/CECS663-2020 [28], the recommendations for material matching are as follows: when using Q355 structural steel, the concrete strength degree should be C30-C60; when using Q390, Q420, and Q460 structural steel, the concrete strength degree should be C60-C90. Such advice is generally sensible but simplistic and ignores the influence of the dimension. The geometric parameters are usually controlled by setting limitations of the diameter-to-thickness ratio or the steel ratio. Therefore, when designers are using those design codes, they often get the parameters that just satisfy the specification rather than the optimal parameters. To further improve the quality and rationality of the design of CCFST columns, in the present study, a framework for the determination of the compatibility of the design parameters of CCFST columns in a straightforward manner was proposed, and explicit recommendations were provided for the parameter matching of axially loaded CCFST stub columns.

This paper is organized as follows: in Section 1, the background and motivation are expressed. Next, in Section 2, an evaluation criterion of the compatibility of CCFST columns as well as a parameter λ are proposed. In Section 3, a framework of determining the λ incorporating four constitutive models of steel and concrete is established, and its validity was verified against a collected experiment database. Using the proposed criterion and framework, the compatibility of design parameters was discussed in Section 4. Section 5 presents an optimization process of CCFST columns with current design code. Section 6 ends the paper with concluding remarks and limitations.

## 2. Evaluation Criterion of the Compatibility of Design Parameters

### 2.1. Composite Actions in CCFST Columns under Axial Load

**Figure 1:**
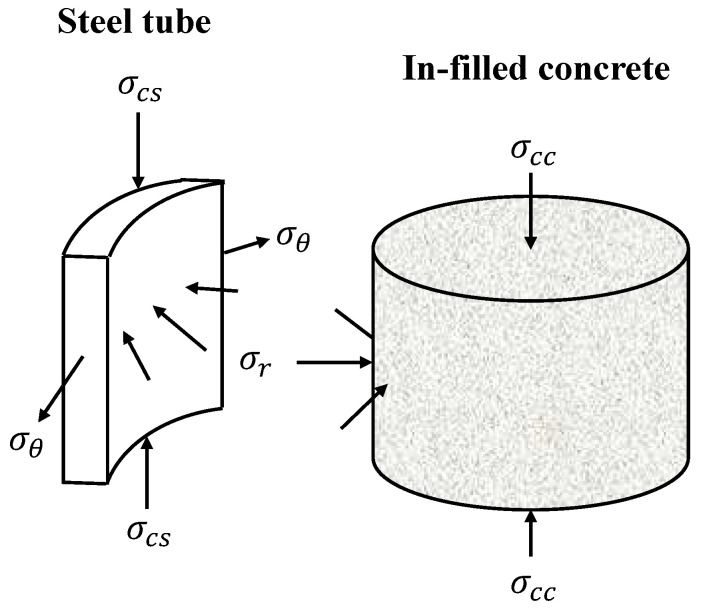
Composite actions in CCFST stub columns under axial load.

The mechanism of the composite actions in CCFST columns under axial load is shown in Figure 1. The in-filled concrete is under the lateral confinement provided by the steel tube, and, therefore, the axial stress $\sigma_{ccu}$ corresponding to the ultimate state of the column is generally larger than its axial compressive strength $f_{c}$. By contrast, the in-filled concrete exerts counteracting pressure on the inner surface of the steel tube, inducing a circumferential stress $\sigma_{\theta }$ in the steel tube. Based on the static equilibrium condition, the relationship of $\sigma_{\theta }\cdot \left(1/\left(\left(2D/t\right)-1\right)\right)=\sigma_{r}$ can be obtained. This circumferential stress $\sigma_{\theta }$ converts the stress condition of the steel tube from uniaxial compressive to biaxial stress. As a result, the axial bearing capacity of steel tube may be weakened due to the composite actions, especially when the tube wall is thick.

In fact, these composite actions usually lead to an enhanced axial bearing capacity of the CCFST columns. However, as revealed by the experimental results reported in the literature [29,30,31], the effectiveness of composite actions is significantly affected by the physical and geometric design parameters. In other words, it is possible to exploit this interaction by selecting design parameters that are reasonably matched.

### 2.2. Numerical Expression of the Effectiveness of the Composite Action in CCFST Columns at the Ultimate State

**Figure 2:**
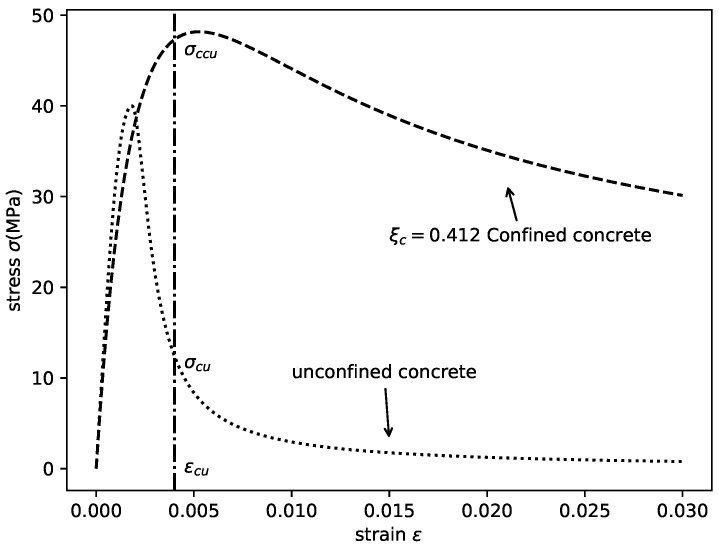
Axial stress of confined concrete and unconfined concrete at the ultimate state of the column ([32,33]).

**Figure 3:**
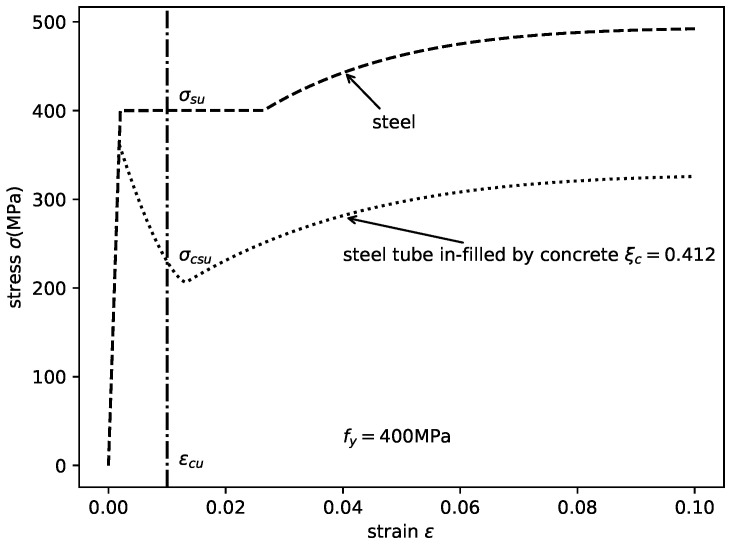
Axial stress of the steel tube in the CCFST columns and structural steel at the ultimate state of the column ([32,34]).

To evaluate the effectiveness of the composite action, a basic assumption was adopted in this study. Namely, the strains of the steel tube and in-filled concrete are consistent under an axial load at the ultimate state of the entire column. Hence, the enhanced axial load $N_{1}$ of the in-filled concrete can be calculated as:

(1) $$N_{1}=\left(\sigma_{ccu}-\sigma_{cu}\right)A_{concrete}$$

where $\sigma_{ccu}$ is the axial stress of the in-filled concrete corresponding to the ultimate strain $\varepsilon_{cu}$ of the entire column; $\sigma_{cu}$ is the axial stress of unconfined concrete when axial strain is $\varepsilon_{cu}$; and $A_{concrete}$ is the area of the cross-section of the in-filled concrete (as shown in Figure 2) [32]. Similarly, the loss of the axial bearing capacity of steel tube $N_{2}$ caused by the interaction with the in-filled concrete can be calculated as:

(2) $$N_{2}=\left(\sigma_{su}-\sigma_{csu}\right)A_{steel}$$

where $\sigma_{su}$ and $\sigma_{csu}$ are the axial stress of steel without or with the interaction of in-filled concrete at the strain of $\varepsilon_{cu}$, respectively, and $A_{steel}$ is the area of the cross-section of the steel tube (as shown in Figure 3) [32]. As a result, the actual enhanced (or reduced) axial bearing capacity caused by the composite action in the CCFST columns can be expressed as:

(3) $$N_{composite}=N_{1}-N_{2}$$

If $N_{composite}$ is larger than zero (in most cases), the composite action exerts a positive effect on the axial bearing capacity of CCFST columns. If $N_{composite}$ is less than zero, the composite action has a negative influence and should be avoided in design. It is clear that the value of $N_{composite}$ is correlated with the design parameters of the CCFST columns. Therefore, to normalize this evaluation criterion and to compare the composite action of the CCFST columns with different design parameters, a composite ratio λ was proposed here:

(4) $$\lambda =N_{composite}/\left(\sigma_{cu}A_{concrete}+\sigma_{su}A_{steel}\right)$$

As defined in the equation, λ represents the proportion of the bearing capacity caused by the composite action to the bearing capacity of the material itself. Therefore, this indicator can be applied to evaluate the effectiveness of the composite action in the CCFST columns. As will be discussed below, the λ values have the upper limit with the variation in the design parameters under certain conditions. Hence, the compatibility of the design parameters of the CCFST columns can be investigated quantitatively.

## 3. Modeling of the Compatibility of CCFST Columns

As depicted in the previous section, once the axial stress–strain relationships of the steel tube and in-filled concrete and the ultimate strain corresponding to the ultimate state of the entire columns are determined, the effectiveness of the composite action in the CCFST columns can be calculated. In this study, four constitutive models that are applicable to a wide range of material strengths and a regression formula of ultimate strain were adopted. It should also be mentioned here that the bond behaviors are neglected in this study, since the Poisson ratio of steel is larger than concrete; at the early stage of loading, the steel will disengages from the concrete. Namely, the failure of bond occurs long before the limit state of the entire columns, which lead to an insignificant influence on the axial loading behaviors.

### 3.1. Stress–Strain Relationships of Steel Tube Confined Concrete

The concrete under the confinement of the steel tube shows an enhanced peak strength and deformation ability compared with unconfined concrete [1,35], as shown in Figure 3. This enhanced behavior is influenced by the diameter to thickness ratio D/t, the compressive strength of concrete $f_{c}$, and yield strength of the outer steel tube $f_{y}$. To capture these confined mechanical behaviors, various uni-axial constitutive models considering the lateral confinement have been proposed, such as Patel et al. [36], Liang and Fragomeni [37], Lai and Varma [38], and Sakino et al. [19], among others. Although these models have been validated in their respective studies, in the compatibility analysis presented herein, a stress–strain relationship that is applicable to the broadest strength range and not affected by the modeling method (element type and mesh size) is required.

The stress–strain relationship proposed by Katwal et al. [32] is adopted herein to express the mechanical behaviors of confined concrete. This model modifies the stress–strain relations proposed by Samani and Attard [39] and thereby is applicable to the fiber element modeling of the axially loaded CCFST columns. It was reported that the numerical results are not sensitive to the mesh size in their fiber modeling, indicating that the model can be used to describe the average axial stress behaviors of in-filled concrete. Moreover, the concrete compressive strength $f_{c}$ in their verification study ranges from 15 MPa to 193 MPa. As a result, this model is appropriate for this study. The effective axial stress–strain curve of the concrete confined by circular steel tube is expressed as:

(5) $$\sigma_{cc}=\left\{\begin{array}{c} \frac{A_{1}\cdot X+B_{1}\cdot X^{2}}{1+\left(A_{1}-2\right)\cdot X+\left(B_{1}+1\right)\cdot X^{2}}\cdot f_{cc}^{{}^{\prime }}\;X\leq 1\;or\;\left(x>1\;and\;\sigma >f_{r}\right)\\ f_{r}\;\;\;\;\;\;\;\;\;\;\;\;X>1\;and\;\sigma \leq f_{r} \end{array}\right.$$

where $X=\varepsilon /\varepsilon_{cc}^{{}^{\prime }}$, $f_{cc}^{{}^{\prime }}$, and $\varepsilon_{cc}^{{}^{\prime }}$ are the peak stress of the stress–strain curve and the corresponding strain, respectively, $f_{r}$ is the residual stress of in-filled concrete after the crushing, and *A* and *B* are the parameters to determine the shape of the stress–strain relationships. These parameters are correlated with the dimension and material characteristics of the outer steel tube.

The confined strength $f_{cc}^{{}^{\prime }}$ is defined as:

(6) $$f_{cc}^{{}^{\prime }}=\left(1+0.2\cdot {\left(\frac{f_{y}}{f_{c}}\right)}^{0.696}+\left(0.9-0.25\cdot {\left(\frac{D}{t}\right)}^{0.46}\right)\cdot \sqrt{\xi }\right)\cdot f_{c}$$

where $f_{c}$ is the strength of unconfined concrete, and ξ is the confinement factor of the CCFST columns, which is defined as $\xi =A_{s}f_{y}/A_{c}f_{c}$.

The strain corresponding to the peak stress $\varepsilon_{cc}^{{}^{\prime }}$ is defined as:

(7) $$\varepsilon_{cc}^{{}^{\prime }}=3000-10.4\cdot f_{y}^{1.4}{\left(f_{c}\right)}^{-1.2}\left[0.73-3785.8{\left(\frac{D}{t}\right)}^{-1.5}\right]$$

where $\varepsilon_{cc}^{{}^{\prime }}$ is in με.

The residual strength $f_{r}$ is expressed as:

(8) $$f_{r}=f_{cc}^{{}^{\prime }}\left[3.5\cdot {\left(\frac{t}{D\cdot f_{c}^{0.7}}\right)}^{0.2}-\frac{0.2}{\xi_{c}^{0.3}}\right]\leq f_{cc}^{{}^{\prime }}$$

The parameters $A_{1}$ and $B_{1}$ determine the shape of the ascending and descending regions of the stress–strain curve, respectively. $A_{1}$ and $B_{1}$ are calculated according to Equations (9) and (10), respectively:

(9) $$A_{1}=\alpha_{1}\frac{E_{c}\varepsilon_{cc}^{{}^{\prime }}}{f_{cc}^{{}^{\prime }}}$$

(10) $$B_{1}=2.15-2.05e^{-\xi_{c}}-0.0076f_{c}\geq -0.75$$

where $\alpha_{1}$ is the correction factor ranging from 1 to 1.3, which is highly correlated to the confinement factor $\xi_{c}$. $\alpha_{1}$ is defined as:

(11) $$\alpha_{1}=1+0.25\cdot \xi_{c}^{\left(0.05+0.25/\xi_{c}\right)}$$

**Figure 4:**
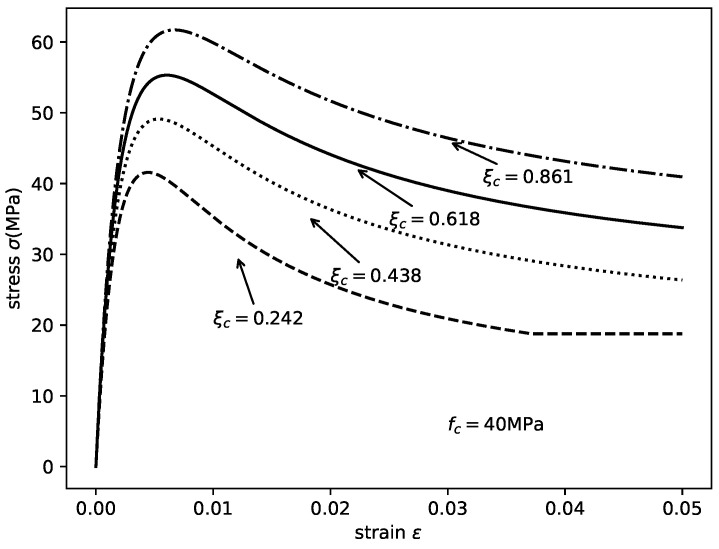
Stress–strain relationships of steel tube confined concrete.

As reported by Katwal et al. [32], the aforementioned parameters were calibrated based on finite element model (FEM) analysis using the regression method. Generally, a good correlation between the calculated parameters and the FEM results were obtained. The obtained stress–strain relation of the steel tube confined concrete with different confinement factor $\xi_{c}$ values is shown in Figure 4.

### 3.2. Axial Stress–Strain Relationships of Unconfined Concrete

The stress–strain relationship of unconfined concrete adopted in this study is proposed by Ding et al. [33]. This model incorporated the ascending branch suggests by Sargin [40] and the descending branch proposed by Zhenhai [41]. The uniaxial stress–strain relation can be described as:

(12) $$\sigma_{c}=\left\{\begin{array}{c} \frac{A_{2}\cdot x+\left(B_{2}-1\right)\cdot x^{2}}{1+\left(A_{2}-2\right)\cdot x+B_{2}\cdot x^{2}}\cdot f_{c}\;x\leq 1\\ \frac{x}{\alpha_{2}{\left(x-1\right)}^{2}+x}\cdot f_{c}\;\;\;x>1 \end{array}\right.$$

where $x=\varepsilon_{c}/\varepsilon_{c0}$. $A_{2}$ represents the ratio of the elastic modulus to peak stress secant modulus of concrete. $B_{2}$ is the degree of attenuation of the concrete elastic modulus. It was found that the aforementioned parameters proposed by Ding et al. [33] predict the stress–strain curves of concrete strength $f_{c}$ less than 60 MPa well, but they provide a slight overestimation of the strength and ductility of high-strength concrete. Hence, some modifications were carried out herein.

**Figure 5:**
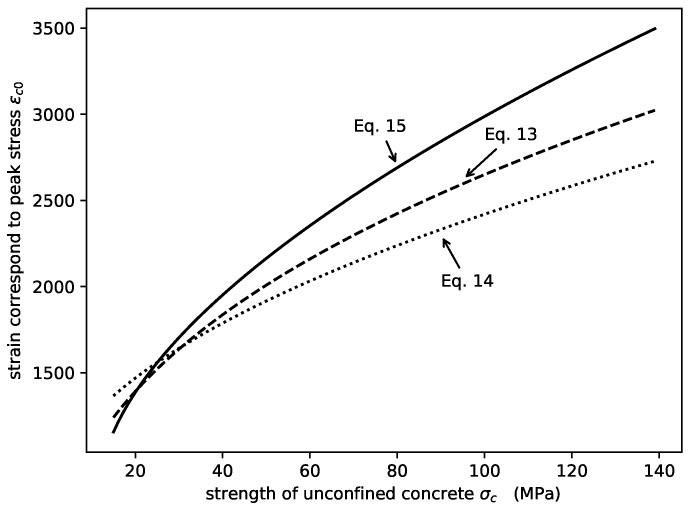
Comparison of $\varepsilon_{c0}$ using different expressions.

Several expressions of the strain corresponding to the peak stress of unconfined concrete $\varepsilon_{c0}$ are available in the literature. In this study, the modified formula of $\varepsilon_{c0}$ proposed in Equation (13) is used. Comparison of the proposed equation and that adopted by Lin et al. [42] (Equation (14)) and De Nicolo et al. [43] (Equation (15)) is shown in Figure 5:

(13) $$\varepsilon_{c0}=420f_{c}^{2/5}$$

(14) $$\varepsilon_{c0}=700+172\sqrt{f_{c}}$$

(15) $$\varepsilon_{c0}=760+\sqrt{\left(0.626f_{cu}-4.33\right)\times 10^{5}}$$

The parameters $A_{2}$ and $B_{2}$ are modified as:

(16) $$A_{2}=6.9f_{cu}^{-11/30}$$

(17) $$B_{2}=1.67{\left(A_{2}-1\right)}^{2}$$

where $f_{cu}$ is the cubic compressive strength obtained from the material testing. The relationship between $f_{c}$ and $f_{cu}$ is given by:

(18) $$f_{c}=0.4f_{cu}^{7/6}$$

**Figure 6:**
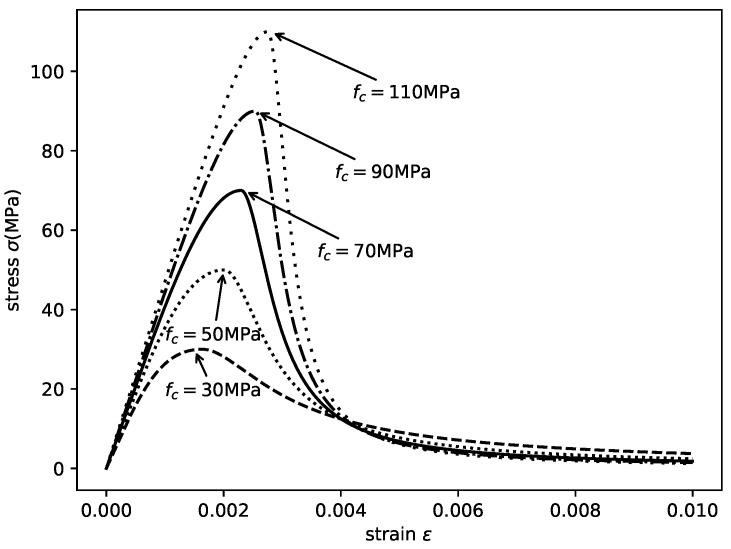
Stress–strain relationships of unconfined concrete.

The modified stress–strain relationship of unconfined concrete is shown in Figure 6. This model can be applied to the analysis of unconfined concrete with the compressive strength up to 140 MPa. Although there are many other stress–strain relationships of unconfined concrete available in the literature, this model shows a good match with the confined concrete model adopted in this study. Additionally, this model suggested a strength that approaches zero smoothly with increasing strain, thus ensuring the numerical stability of the presented compatibility analysis.

### 3.3. Size Effect of Steel Tube Confined Concrete

The size effect of concrete may have an impact on the compatibility of the design parameters of CCFST columns. In this study, the modifications proposed by Lin et al. [44] are adopted to consider the size effect of the steel tube confined concrete. The strain corresponding to the peak point is modified as:

(19) $$\varepsilon_{cc}^{{}^{\prime }}/\varepsilon_{c0}=1+4200D^{-0.9}\sqrt{f_{y}}e^{-0.04f_{c}}{\left(D/t\right)}^{-0.8}$$

The influence of the size effect on the peak stress $f_{cc}^{{}^{\prime }}$ and residual stress $f_{r}$ is considered by introducing the reduction factor $R_{d,cc}$ and $R_{d,cr}$, which can be expressed as:

(20) $$R_{d,cc}=1.67^{{\left(1+\eta \right)}^{-8}}D_{c}^{-(0.112*0.0005^{\eta })}$$

(21) $$R_{d,cr}=-0.13e^{-0.01f_{c}}D^{0.31}+1.48e^{-0.002f_{c}}$$

(22) $$\eta =\frac{2t}{D-2t}\frac{f_{y}}{f_{c}}$$

It should be noted here that, in the model of Lin et al. [44], the size effect on the residual stress is considered by multiplication of $R_{d,cr}$ by a function of $f_{c}$. However, in the model of Katwal et al. [32], $f_{r}$ is expressed as a function of $f_{cc}^{{}^{\prime }}$. To avoid repeated consideration of the size effect in residual stress $f_{r}$, the modified peak stress and residual stress are expressed as:

(23) $$f_{cc,size}=R_{d,cc}f_{cc}^{{}^{\prime }}$$

(24) $$f_{r,size}=\frac{R_{d,cr}}{R_{d,cc}}f_{r}$$

**Figure 7:**
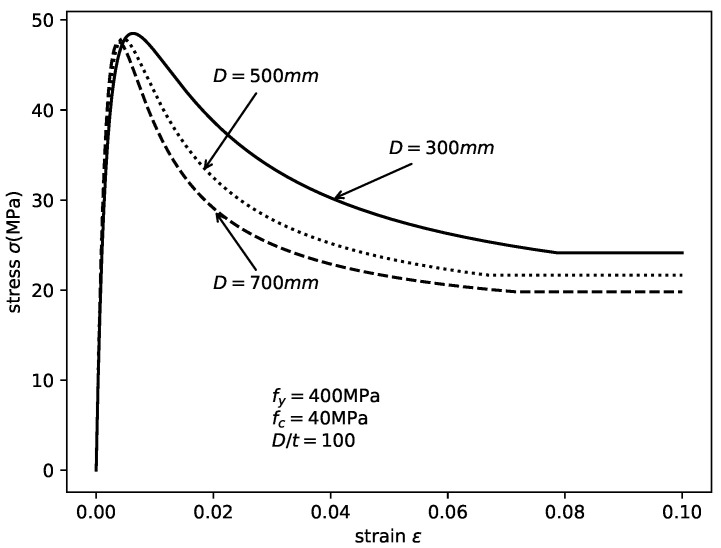
Size effect on the stress–strain relation of confined concrete.

The modified stress–strain relation of in-filled concrete for considering the size effect is shown in Figure 7. It is observed that, with the same $f_{y}$, $f_{c}$ and D/t ratio, the peak stress and residual stress of in-filled concrete decrease with increasing cross-sectional size. The strain corresponding to the peak stress $\varepsilon_{cc}^{{}^{\prime }}$ also decreases.

### 3.4. Axial Stress–Strain Relationships of the Steel Tube in Axially Loaded CCFST Columns

According to the findings reported by Lin et al. [44], there is almost no size effect on the peak stress $f_{y}^{{}^{\prime }}$ and the corresponding strain $\varepsilon_{y}^{{}^{\prime }}$ of the steel tube and only a minor impact on the critical stress $f_{cr}^{{}^{\prime }}$ and strain $\varepsilon_{cr}^{{}^{\prime }}$. Moreover, the axial strain of the steel tube corresponding to the ultimate state of the columns $\varepsilon_{cu}$ is lower than the critical strain $\varepsilon_{cr}^{{}^{\prime }}$ in most cases. Therefore, the size effect of the outer steel tube is ignored in this study.

The model proposed by Katwal et al. [32] is adopted herein to express the axial stress–strain relationships of the steel tube influenced by in-filled concrete:

(25) $$\sigma_{cs}=\left\{\begin{array}{c} E_{s}\varepsilon_{s}\;\;\;\;\;\;0\leq \varepsilon_{s}<\varepsilon_{y}^{{}^{\prime }}\\ f_{cr}^{{}^{\prime }}-\left(f_{cr}^{{}^{\prime }}-f_{y}^{{}^{\prime }}\right)\cdot {\left(\frac{\varepsilon_{cr}^{{}^{\prime }}-\varepsilon_{s}}{\varepsilon_{cr}^{{}^{\prime }}-\varepsilon_{y}^{{}^{\prime }}}\right)}^{\psi }\;\varepsilon_{y}^{{}^{\prime }}\leq \varepsilon_{s}<\varepsilon_{cr}^{{}^{\prime }}\\ f_{u}^{{}^{\prime }}-\left(f_{u}^{{}^{\prime }}-f_{cr}^{{}^{\prime }}\right)\cdot {\left(\frac{\varepsilon_{u}-\varepsilon_{s}}{\varepsilon_{u}-\varepsilon_{cr}^{{}^{\prime }}}\right)}^{p}\;\varepsilon_{cr}^{{}^{\prime }}\leq \varepsilon_{s}<\varepsilon_{u}\\ f_{u}^{{}^{\prime }}\;\;\;\;\;\;\;\;\varepsilon_{s}\geq \varepsilon_{u} \end{array}\right.$$

where $f_{y}^{{}^{\prime }}$ and $\varepsilon_{y}^{{}^{\prime }}$ are the peak stress and the corresponding strain of the stress–strain curve, respectively, $E_{s}$ is the Young’s modulus of the steel, $\sigma_{cs}$ and $\varepsilon_{s}$ are the axial stress and strain of the steel tube in the CCFST columns, respectively, $f_{u}^{{}^{\prime }}$ and $\varepsilon_{u}$ are the ultimate stress and strain, respectively, ψ is the strain softening exponent, and *p* is the strain-hardening exponent.

The peak stress $f_{y}^{{}^{\prime }}$ of the stress–strain curve of the steel tube in the CCFST columns is defined as:

(26) $$f_{y}{}^{\prime }/f_{y}=1.02-0.01\cdot {\left(\frac{\varepsilon_{y}}{\varepsilon_{c0}}\right)}^{1.5}{\left(\frac{D}{t}\right)}^{0.5}\leq 1$$

where $f_{y}$ is the yield strength of steel, and $\varepsilon_{y}$ is the strain corresponding to $f_{y}$, which is expressed as $\varepsilon_{y}=f_{y}/E_{s}$.

The critical stress $f_{cr}^{{}^{\prime }}$ and critical strain $\varepsilon_{cr}^{{}^{\prime }}$ are defined as:

(27) $$f_{cr}^{{}^{\prime }}=f_{y}\cdot e^{\left(-0.39+0.1\xi_{c}+0.06\ln\left(\xi_{c}\right)/\xi_{c}^{2}\right)}>0\;and\leq \;f_{y}^{{}^{\prime }}$$

(28) $$\varepsilon_{cr}^{{}^{\prime }}=\varepsilon_{y}\left[28-0.07\xi_{c}-\frac{12}{\xi_{c}^{0.2}}-0.13f_{y}^{0.75}{\left(\frac{t}{D\cdot {\left(f_{c}^{{}^{\prime }}\right)}^{0.7}}\right)}^{0.07}\right]\;\varepsilon_{cr}^{{}^{\prime }}\geq \varepsilon_{y}\;and\;\varepsilon_{cr}^{{}^{\prime }}\leq \varepsilon_{u}$$

The ultimate stress $f_{u}^{{}^{\prime }}$ and strain $\varepsilon_{u}$ are expressed as:

(29) $$f_{u}^{{}^{\prime }}=f_{y}\left[6.8-0.013\xi_{c}-\frac{3.5}{\xi_{c}^{0.15}}-1.3f_{y}^{0.25}{\left(\frac{t}{D\cdot {\left(f_{c}^{{}^{\prime }}\right)}^{0.7}}\right)}^{0.15}\right]\;f_{u}^{{}^{\prime }}\geq f_{cr}^{{}^{\prime }}\;and\;f_{u}^{{}^{\prime }}\leq f_{u}$$

(30) $$\varepsilon_{u}=\left\{\begin{array}{c} 100\varepsilon_{y}\;\;\;\;\;f_{y}\leq 300\;\mathrm{MPa}\\ \left[100-0.15(f_{y}-300)\right]\varepsilon_{y}\;300<f_{y}\leq 800\;\mathrm{MPa}\\ \left[25-0.1(f_{y}-800)\right]\varepsilon_{y}\;800<f_{y}\leq 960\;\mathrm{MPa} \end{array}\right.$$

The strain-softening exponent ψ is defined as a constant value of 1.5. The strain-hardening exponent *p* is expressed as:

(31) $$p=E_{p}\left(\frac{\varepsilon_{u}-\varepsilon_{cr}^{{}^{\prime }}}{f_{u}^{{}^{\prime }}-f_{cr}^{{}^{\prime }}}\right)$$

where $E_{p}$ is the initial modulus of elasticity at the onset of strain-hardening, which is taken as $0.02E_{s}$.

**Figure 8:**
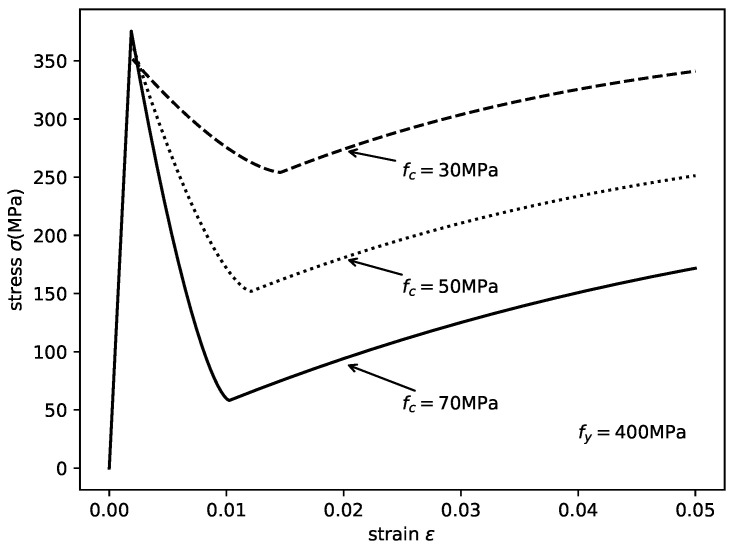
Axial stress–strain relationships of the steel tube in the CCFST columns with different concrete strengths.

As shown in Figure 8, with different strengths of in-filled concrete, the axial stress–strain relation of the steel tube changed significantly. The critical stress, residual stress, and ultimate stress decreased with increasing concrete strength. The critical strain also decreased. However, the peak stress $f_{y}^{{}^{\prime }}$ increased slightly.

### 3.5. Stress–Strain Relationships of Steel Tubes

The stress–strain relationships of structural steel are used in this study to determine the loss of the axial bearing capacity of the steel tube caused by the interaction with in-filled concrete. The model proposed by Tao et al. [34] is adopted here since its form is consistent with the adopted stress–strain relationships of the steel tube in the CCFST columns. The stress–strain relationships of structural steel are expressed as:

(32) $$\sigma_{s}=\left\{\begin{array}{c} E_{s}\varepsilon_{s}\;\;\;\;\;\;0\leq \varepsilon_{s}<\varepsilon_{y}\\ f_{y}\;\;\;\;\;\;\varepsilon_{y}\leq \varepsilon_{s}<\varepsilon_{p}\\ f_{u}-\left(f_{u}-f_{y}\right)\cdot {\left(\frac{\varepsilon_{u}-\varepsilon_{s}}{\varepsilon_{u}-\varepsilon_{p}}\right)}^{\theta }\;\;\varepsilon_{p}\leq \varepsilon_{s}<\varepsilon_{u}\\ f_{u}\;\;\;\;\;\;\;\varepsilon_{s}\geq \varepsilon_{u} \end{array}\right.$$

where $f_{u}$ is the ultimate strength of structural steel, $\varepsilon_{p}$ is the strain at the onset of strain hardening, and θ is the strain-hardening exponent.

The consistent definition of $\varepsilon_{u}$ and $\varepsilon_{y}$ is adopted with the stress–strain relation of the steel tube in the CCFST columns. The ultimate strength of structural steel $f_{u}$ is defined as:

(33) $$f_{u}=\left\{\begin{array}{c} \left[1.6-2\times 10^{-3}\left(f_{y}-200\right)\right]f_{y}\;200\;\mathrm{MPa}\leq f_{y}\leq 400\;\mathrm{MPa}\\ \left[1.2-3.75\times 10^{-4}\left(f_{y}-400\right)\right]f_{y}\;400\;\mathrm{MPa}\leq f_{y}\leq 800\;\mathrm{MPa}. \end{array}\right.$$

**Figure 9:**
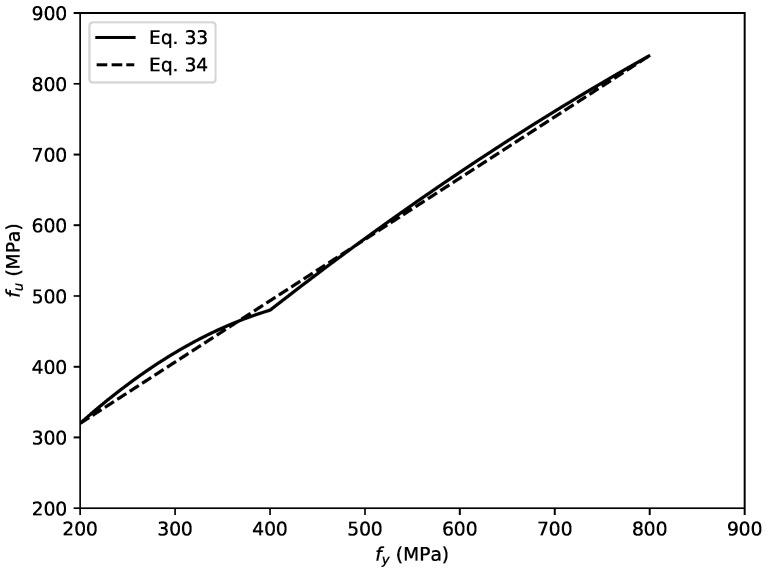
Relationships of ultimate stress $f_{u}$ and yield strength $f_{y}$.

However, as shown in Figure 9, a discontinuity is present when $f_{y}=400$ MPa. This case may lead to an unsmooth variation in λ with changes in $f_{y}$. Hence, we modified the expression of $f_{u}$ as a linear function of $f_{y}$ (Equation (34)). It is found from Figure 9 that the modified function almost coincides with Equation (33):

(34) $$f_{u}=\frac{13}{15}f_{y}+\frac{440}{3}$$

The strain corresponding to the onset of strain-hardening $\varepsilon_{p}$ is expressed as:

(35) $$\varepsilon_{p}=\left\{\begin{array}{c} 15\varepsilon_{y}\;\;\;\;\;f_{y}\leq 300\;\mathrm{MPa}\\ \left[15-0.018(f_{y}-300)\right]\varepsilon_{y}\;300\;MPa\leq f_{y}\leq 800\;\mathrm{MPa} \end{array}\right.$$

The strain-hardening exponent θ is defined as:

(36) $$\theta =E_{p}\cdot \left(\frac{\varepsilon_{u}-\varepsilon_{p}}{f_{u}-f_{y}}\right)$$

**Figure 10:**
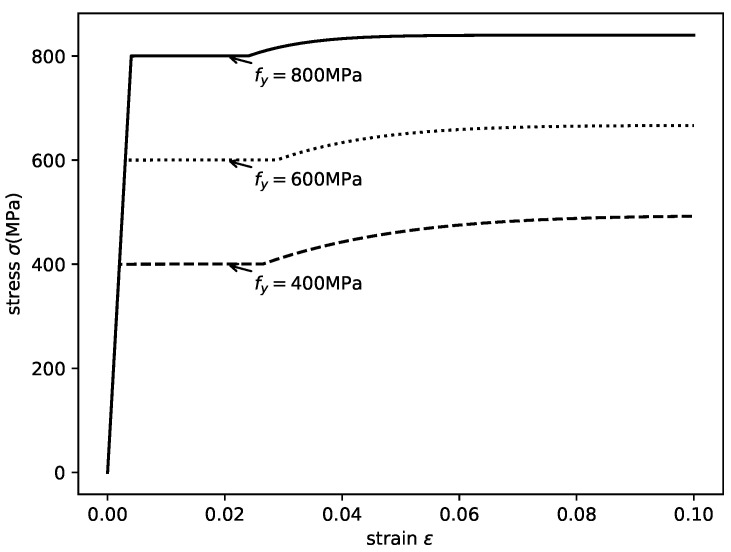
Axial stress–strain relationships of structural steel.

The adopted stress–strain relation of the steel is shown in Figure 10.

It should be noted here that the local buckling strongly affects the axial strength of the hollow steel tube. Since we are comparing the axial stress of steel tube with or without in-filled concrete, the influence of local buckling in the hollow steel tube should be considered. For this purpose, a reduction factor introduced by Grimault and Janss [45] is adopted here to modify the yield strength of structural steel in the hollow steel tubes with different diameter-to-thickness ratios:

(37) $$K_{v}=\left\{\begin{array}{c} 1.0\;\;\;\;\;\;\;\frac{D}{t}\leq \sqrt{\frac{8E_{s}}{f_{y}}}\\ K_{v}=\frac{D}{2t}\frac{f_{y}}{32,960}+\frac{21,190}{(\frac{D}{2t}f_{y})+21,190}\;\frac{D}{t}>\sqrt{\frac{8E_{s}}{f_{y}}} \end{array}\right.$$

The modified yield strength of the hollow steel tube is given by:

(38) $$f_{y,hollow}=f_{y}/K_{v}$$

### 3.6. Determination of Axial Strain $\varepsilon_{cu}$ of the Ultimate State of the Column

The determination of axial strain $\varepsilon_{cu}$ corresponding to the ultimate state of the CCFST columns is essential for the compatibility analysis presented in this study. Wang et al. [24] proposed an equation for the prediction of $\varepsilon_{cu}$ based on the regression analysis of FEM results, which can be expressed as:

(39) $$\varepsilon_{cu}=3000-10.4f_{y}^{1.4}{\left(f_{c}^{{}^{\prime }}\right)}^{-1.2}\left[0.73-3785.8{\left(D/t\right)}^{-1.5}\right]\leq 10,000\mu \varepsilon$$

where $f_{c}^{{}^{\prime }}$ is the cylinder compressive strength of concrete. Its conversion relation with $f_{cu}$ is defined by:

(40) $$f_{c}^{{}^{\prime }}=\left\{\begin{array}{c} 0.8f_{cu}\;\;\;\;\;f_{cu}\leq 50\;\mathrm{MPa}\\ f_{cu}-10\;\;\;300\;\mathrm{MPa}\leq f_{cu}>50\;\mathrm{MPa} \end{array}\right.$$

**Figure 11:**
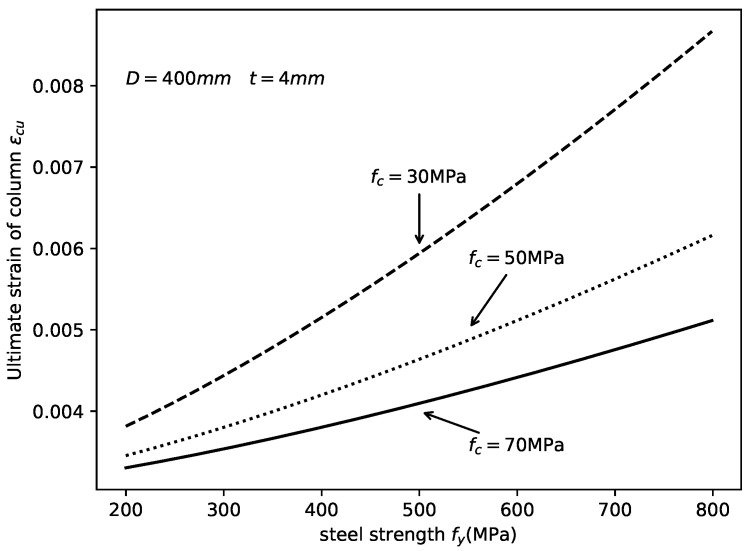
Effect of material strength on $\varepsilon_{cu}$.

**Figure 12:**
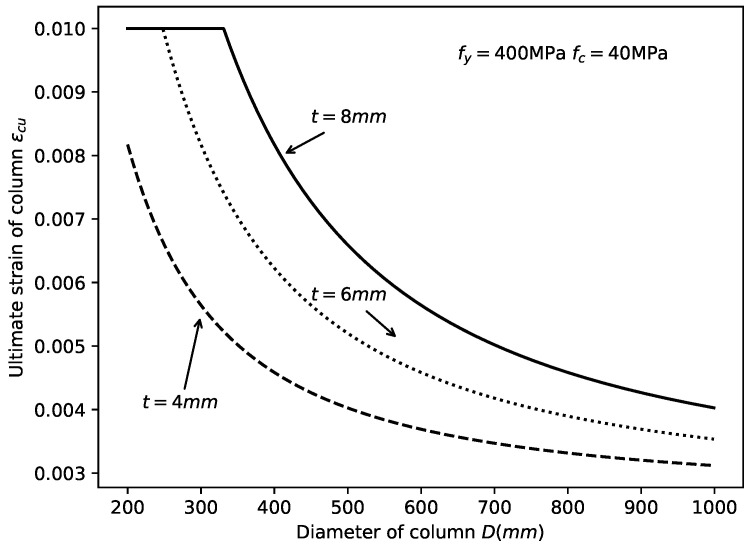
Effect of dimensions on $\varepsilon_{cu}$.

The variation in $\varepsilon_{cu}$ with the material strength is shown in Figure 11. It is concluded here that, with the increase in the steel yield strength $f_{y}$, the ultimate strain of the CCFST column increased. Moreover, with the increase in the concrete compressive strength $f_{c}$, the $\varepsilon_{cu}$ decreased. The influence of dimensions on the ultimate stain is shown in Figure 12. The figure indicates that the ultimate strain decreased with increasing column diameter and decreasing tube wall thickness.

### 3.7. Verification of the Modeling of CCFST Columns Subjected to Axial Loading

**Figure 13:**
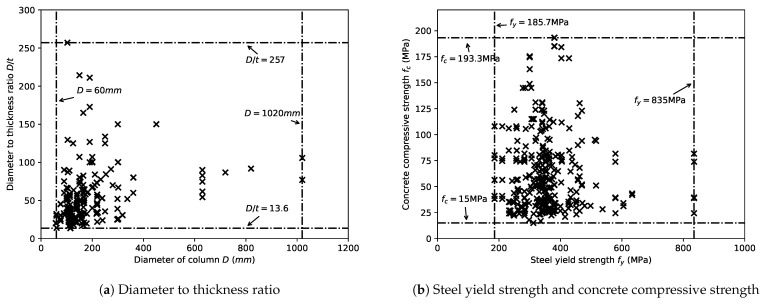
General scope of the design parameters of the axially loaded CCFST columns.

A comprehensive experimental database of axially loaded CCFST stub columns was established [46]. In total, 478 specimens were included with a wide range of D/t ratio, steel strength $f_{y}$, and concrete strength $f_{c}$ values. The data for these test specimens were collected from the literature [16,19,29,30,47,48,49,50,51,52,53,54,55,56,57,58,59,60,61,62,63,64,65,66,67,68,69,70,71,72,73,74,75,76,77,78,79,80,81]. The detailed information regarding the collected specimens and the corresponding references is provided in Table 1. An investigation of this database was performed herein to capture the general scope of the design parameters of CCFST columns. As shown in Figure 13a, the covered range of the D/t ratio is 13.6–257. The concrete strength and steel strength vary from 15 MPa to 193.3 MPa and from 185.7 MPa to 835 MPa, respectively, as shown in Figure 13b. As a result, the range of the design parameters investigated in this study is defined as follows: D/t ratio from 10 to 300, steel strength from 180 MPa to 900 MPa, and concrete strength from 15 MPa to 140 MPa. We note that the upper limit of concrete strength is less than the investigated value. This is because, although it was verified that the model suggested by Katwal et al. [32] of the steel tube confined concrete can be applied to ultrahigh-strength concrete (UHSC), the uniaxial stress–strain relation of unconfined concrete adopted in this study Ding et al. [33] is only applicable to concrete strength values lower than 140 MPa.

The stress–strain relationships adopted in this study have been verified to be reasonable in previous studies. However, in this study, modifications were performed on these models, and their combined use is questionable. Hence, the experiment database was employed here to verify the validity of the adopted method. The axial bearing capacity of the CCFST columns were calculated by the following steps:

- Step 1. Calculate the ultimate strain corresponding to the ultimate state of the columns $\varepsilon_{cu}$, area of steel tube $A_{s}$, and area of in-filled concrete $A_{c}$.
- Step 2. Substitute the $\varepsilon_{cu}$ into the stress–strain relationships of confined concrete and the steel tube in the CCFST columns. Calculate the $\sigma_{csu}$ and $\sigma_{cu}$.
- Step 3. Calculate the axial bearing capacity of the CCFST columns by:
  (41) $$N_{cu}=A_{s}\sigma_{csu}+A_{c}\sigma_{cu}$$

**Figure 14:**
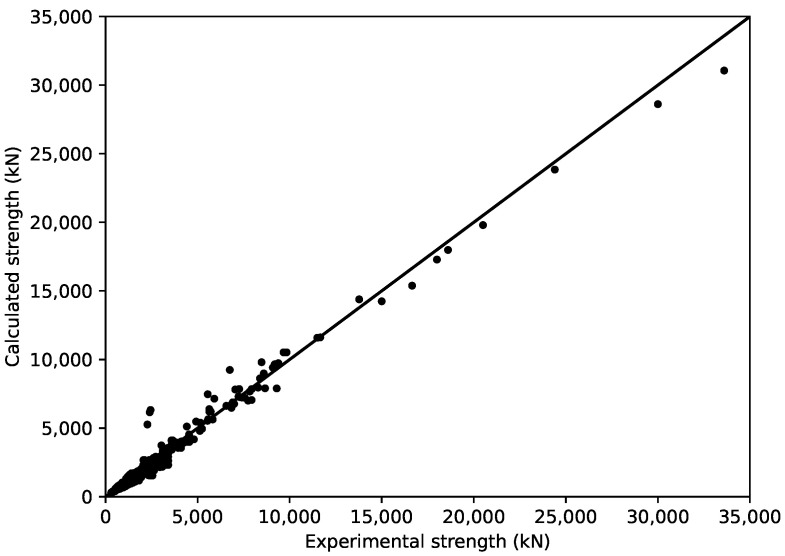
Calculated axial bearing capacity versus the experimental results.

**Figure 15:**
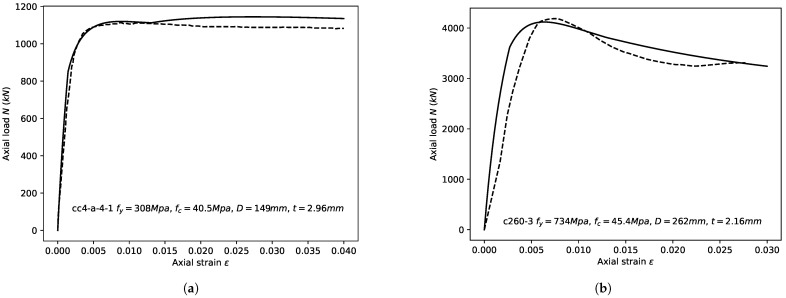
Comparison of calculated axial load–strain curves and the experimental results. (**a**) cc4-a-4-1 [19]; (**b**) c260-3 [82].

The performance of adopted modeling methods in terms of the axial bearing capacity is shown in Figure 14. The figure indicates that the aforementioned method provided a good prediction of the axial bearing capacity of the CCFST columns. Moreover, the adopted formula of $\varepsilon_{cu}$ matched the adopted stress–strain relationships of confined concrete and the steel tube well. The axial load–strain relation of the specimens presented by Sakino et al. [19] and Zhou et al. [82] were also compared with the calculated results, as shown in Figure 15. The calculated axial load–strain curves coincide well with the test results, indicating the validity of the adopted stress–strain relationships.

## 4. Compatibility of the Design Parameters of Axially Loaded CCFST Stub Columns

**Figure 16:**
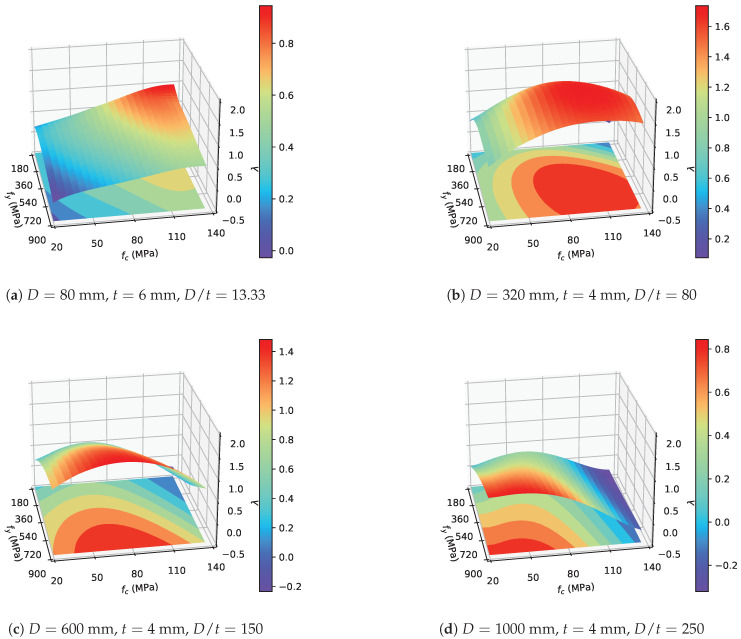
Variation in the composite ratio λ with different material strengths.

The effect of composite actions in the axially loaded CCFST stub columns can be numerically evaluated by the composite ratio λ using the aforementioned method. As shown in Figure 16, with a constant diameter and tube wall thickness, the variation in λ can be represented by a curved plane in three-dimensional space with material strengths and composite ratios as axes. When the diameter-to-thickness ratio is extremely small (D=80 mm, t=6 mm, and D/t=13.33, as shown in Figure 16a), λ monotonically decreases with increasing steel yield strength and decreasing concrete compressive strength. The highest composite ratio was obtained using low-strength steel tube and ultrahigh-strength concrete. Moreover, the lowest λ values were obtained using high-strength steel and low-strength concrete. This phenomenon may contradict the current understanding of the confinement effect in CCFST columns. However, previous studies have invariably emphasized the confinement effect exerted by the steel tube while ignoring the utilization efficiency of the steel tube. Figure 16a reveals that, when the D/t ratio is very small, the contribution of the confinement effect to the axial bearing capacity is no longer dominant so that blindly pursuing a strong confinement effect is uneconomical and unreasonable. It should be also noted here that the overall value of λ is low (under 1.0) when the D/t ratio is very small, which should be avoided in design.

For moderate D/t ratios (D=320 mm, t=4 mm, and D/t=80, as shown in Figure 16b, and D=600 mm, t=4 mm, and D/t=150, as shown in Figure 16c), the distribution of λ in this three-dimensional space formed a shell-like surface. The overall λ values are higher than those of the CCFST columns with a small D/t ratio. Moreover, these figures indicate that there is an optimal match of material strength that maximizes the composite ratio λ within the investigated scope for a moderate D/t ratio. It should be also noted here that the combined use of low-strength steel and high-strength concrete should be avoided because the composite action either barely contributes or contributes negatively to the axial bearing capacity of the columns.

If the D/t ratio is extremely large (D=1000 mm, t=4 mm, and D/t=250, as shown in Figure 16d, the curved plane shows a roughly opposite trend from that of Figure 16a. The highest λ values were obtained using high-strength steel and low-strength concrete. Moreover, the usage of high-strength concrete is undesired in this case. The overall performance of the composite ratio is also poor. Based on the aforementioned analysis, qualitative conclusions can be drawn. First, an excessive or very small D/t ratio should be avoided in design. Second, with a proper dimension, the combined use of low-strength steel and high-strength concrete should be avoided.

**Figure 17:**
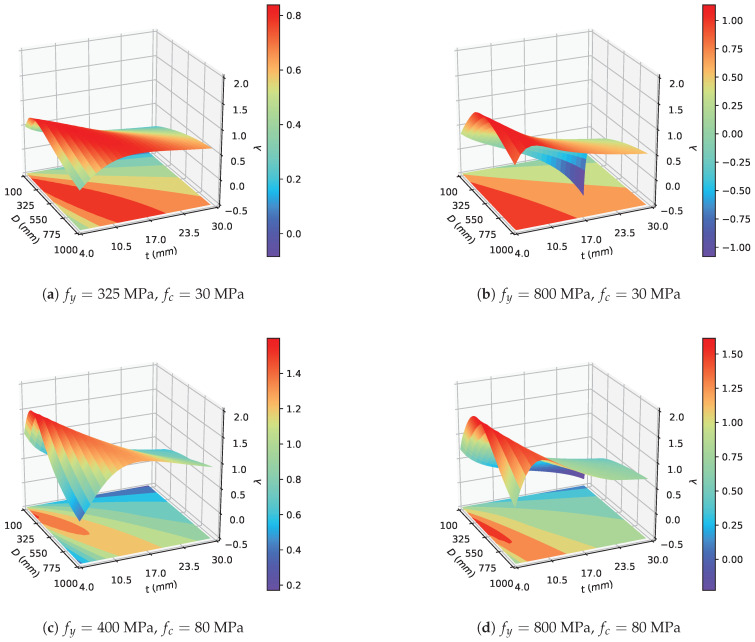
Variation in the composite ratio λ with different dimensions.

**Figure 18:**
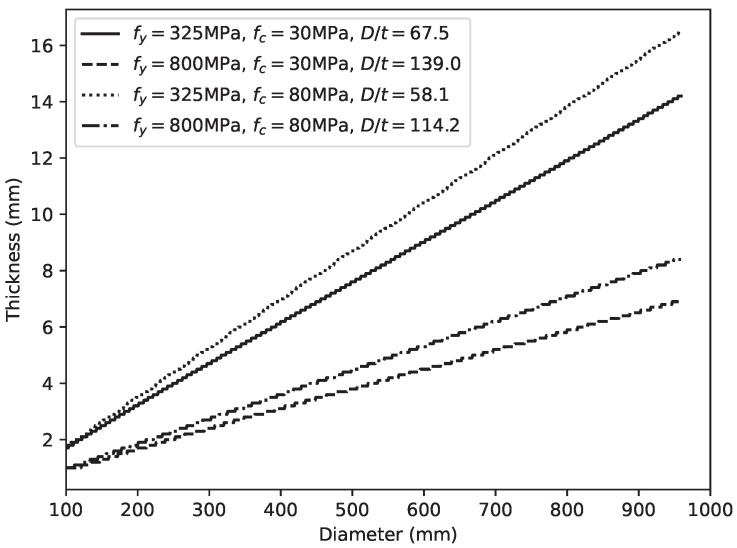
Optimal geometric parameter matches corresponding to different material strengths.

To further investigate the compatibility of the design parameters of the CCFST stub columns subjected to an axial load, the variation in λ with different geometric parameters and certain matches of material strength was plotted, as shown in Figure 17. Among several material strength matches, low-strength steel with low-strength concrete ($f_{y}=325$ MPa and $f_{c}=30$ MPa, as shown in Figure 17a), presented the worst compatibility. Conversely, the use of high-strength steel and high-strength concrete ($f_{y}=800$ MPa and $f_{c}=80$ MPa, as shown in Figure 17d) leads to the highest composite ratio. Moreover, when we mapped the ridges of the surface of λ onto the plane with geometric parameters as its axis, a straight line was obtained (as shown in Figure 18). This result indicated that there is a theoretically optimal value of the D/t ratio corresponding to a certain match of material strength. Following this insight, the optimal D/t ratios of material strength matches including common strength grades of structural steel and concrete were calculated, as presented in Table 2. It can be summarized that a moderate D/t ratio is generally recommended (40.74≤D/t≤149.68). Moreover, the optimal D/t ratio follows the trend of increasing with the steel yield strength $f_{y}$ and decreasing with the compressive strength $f_{c}$ of the in-filled concrete. Based on the data in Table 2, designers can adjust the design parameters to make better use of the composite actions in CCFST columns. If the selected material strength is not listed in the table, the interpolation method can be used to find an approximate optimal value of the D/t ratio.

## 5. Optimization of the Design Parameters of Axially Loaded CCFST Columns

In the previous section, the compatibility of design parameters of the axially loaded CCFST stub columns were investigated, and practical design suggestions were proposed in Table 2. In this section, the proposed composite ratio λ was applied to perform the optimization design of the CCFST columns under certain design scenarios. In practical design, the diameter of the column is usually determined by the required bearing capacity and geometric factors. With the predetermined columns diameter and required bearing capacity, the selection of other design parameters can be described as an optimization problem with the composite ratio as the objective function. The numerical expression of the optimization design can be expressed as:

(42) $$\begin{array}{c} \max(\lambda )\;\;\;\;\\ s.t.\;\left\{\begin{array}{c} \;N_{upper}\geq N_{u}\geq N_{required}\\ \;D=D_{predetermined}\\ \;t_{min}\leq t\leq 30\;\left(mm\right)\\ \;200\;\left(MPa\right)\leq f_{y}\leq 900\;\left(MPa\right)\\ \;20\;\left(MPa\right)\leq f_{c}\leq 100\;\left(MPa\right) \end{array}\right. \end{array}$$

where $N_{required}$ is the required axial bearing capacity according to the design specification, and $N_{u}$ is the calculated axial bearing capacity of CCFST columns. $N_{u}$ can be calculated by the existing design method, which is applicable to a wide range of design parameters. $N_{upper}$ is the upper limit of the axial bearing capacity and is defined as $1.1N_{required}$, $D_{predetermined}$ is the predetermined diameter of CCFST column, and $t_{min}$ is the lower limit tube wall thickness according to adopted design specification. In the engineering practice, very high steel pipe wall thickness often results in poor welding quality. Hence, the upper limit of the tube wall thickness t=30 mm was selected. The range of material strength was selected according to the current achievable structural steel and concrete.

For instance, we need to design an axially loaded CCFST stub column with a diameter of 400 mm according to T/CECS663-2020 [28]. The required axial bearing capacity of the columns is 10,000 kN. The required lower limit of tube wall thickness is defined as:

(43) $$t\geq D/(150\left(\frac{235}{f_{y}}\right))$$

where *D* and *t* are in mm, and $f_{y}$ is in MPa. The axial bearing capacity of the CCFST columns can be calculated as:

(44) $$N_{u}=\left(1.14+1.02\xi \right)f_{c}A_{sc}$$

where ξ is the confinement factor as defined in Equation (6). $A_{sc}$ is the sum of the area of $A_{s}$ and $A_{c}$.

Since the volume of the optimization problem is small, the problem can be solved by the multiple loop method. Considering the engineering practice, the interval of the design parameter to be solved were selected as 0.1 mm, 20 MPa, and 1 MPa for the tube wall thickness, steel yield strength, and compressive strength of concrete, respectively. The optimized design parameters are $f_{y}=440$ MPa, $f_{c}=56$ MPa, and t=5.0 mm. The corresponding axial bearing capacity is 10,947.3 kN, and the composite ratio λ is 1.359.

## 6. Conclusions

In the presented study, the compatibility of the design parameters of the axially loaded CCFST stub columns was quantitatively investigated in terms of the contribution of composite actions to the axial bearing capacity. The main works and findings presented in this study are as follows:

1. A parameter, the composite ratio λ, was proposed as an indicator to represent the effectiveness of the composite actions. A numerical framework of the determination of λ was established, making use of a series of existing widely recognized constitutive models of structural steel and concrete. Some modifications were carried out on these models to ensure the numerical stability of the presented framework. The rationality of the combined use of these models was verified.
2. Using the proposed method, the compatibility of the different design parameter matches was studied. The results show that the D/t ratio of circular CFST columns should not be too big or small. Meanwhile, the combined usage of low-strength steel and high-strength of concrete should be avoided. For the application of design, a table contains the theoretical optimal values of the diameter-to-thickness ratio corresponding to the different matches of material strengths were presented.
3. An optimization design of the axially loaded CCFST stub columns incorporating the proposed method and T/CECS663-2020 [28] was performed. The proposed numerical framework can be applied to optimize the design parameters of axially compressed CFST columns. It should be noted here that the proposed method can be applied with other existing design specifications as well.

Last but not least, the proposed method can only be applied to optimize the axially loaded CFST columns, since the adopted constitutive relations are applicable only in this case. The adopted constitutive models in the proposed method are applicable to a wide range of design parameters, and their validity has been extensively verified by experimental and numerical studies. Therefore, design suggestions presented in this study are considered to be reasonable. However, further improvement in the modeling of the effectiveness of composite actions can be performed from the following two perspectives. The strain of the steel tube and in-filled concrete corresponding to the ultimate state of the column can be calculated more precisely. The influence of the load path effect, namely, the passively confined strain–stress relations, can be applied to further improve the accuracy of the proposed method.

## Figures and Tables

**Table 1:** Test data available in the literature.

Author	No.	*D* (mm)	*t* (mm)	*L* (mm)	$f_{s}$ (MPa)	$f_{c}$ (MPa)	$N_{\exp}$ (kN)
Abed et al. [47]	6	114,167	3.1–5.6	250,350	300	44,60	1042–1873
de Oliveira et al. [48]	16	114.3	3.4,6	343,572	287.3–343	32.7–105.5	749–1943
Fam et al. [49]	1	152	3.1	457	347	55	2389
Gardner and Jacobson [16]	12	76.4–152.6	1.68–4.93	152.3–304.9	415.1–633.4	20.9–43.4	355–2913
Gardner [50]	11	168.8,169.3	2.62–5	305	260.7–333.2	17.9–36.6	1220–2009
Giakoumelis and Lam [51]	13	114.09–115.04	3.8–5	300	343,365	25.1,89.1	948–1787
Gupta et al. [52]	12	89.32,112.56	2.7,2.9	340	360	25.2–40	610–822
Han and Yao [53]	12	100,200	3	300,600	303.5	50	708–2383
Han et al. [54]	26	60–250	1.87,2	180–750	282,404	75.6,80.3	312–4800
He et al. [55]	21	150–273	3,4.5	465–704	318.3–380	31.3–65.8	1470–3689
Huang et al. [56]	3	200–300	2–5	840	265.8–341.7	27.2,31.2	2017–3025
Johansson and Gylltoft [57]	1	159	4.8	650	433	64.5	2150,2220
Kato [58]	12	297–301.5	4.5–11.88	895.2–904.5	347.9–471.4	26.6–79.1	3851–9388
Liu et al. [59]	2	133.05,138.5	3.2–6.2	399	351	75.1	1890
O’Shea and Bridge [60]	15	165,190	0.9–2.8	577.5	185.7–363.3	41–108	1350–3360
Saisho et al. [61]	29	101.6,139.8	2.37–2.99	304.8,419.4	341–462.6	24.4–130.2	676–2165
Sakino et al. [19]	36	108–450	3–6.5	324–1350	283–835	24.4–81.7	941–13,776
Schneider [62]	3	140,141	3–6.7	602,616	285–537	23.8,28.2	881–2363
Tan [63]	20	125–133	1–7	378–465	232–429	45.8–106.1	1239–3370
Tang et al. [64]	27	92–250	1.5–10	276–1480	233.2–433.2	24.2–60.9	505–5135
Wang and Qian [65]	14	114.3–219.0	2.34–3.9	456–876	315–373	57.1,70.6	926–3602
Xue et al. [66]	3	219	3-5	700	313	53.8	2647–3218
Yamamoto et al. [67]	13	101.4–318.5	3.02–10.38	304.2–955.5	339–452	22.3–50.1	649–8289
Yu et al. [68]	28	149–165	1–8	500	338–438	74.1,78.5	1372–3330
Yu et al. [69]	6	165–219	2.7–4.8	510,650	350	38.6–67.9	1560–3400
Yu et al. [70]	4	100	1.9	300	404	111.7	1085–1170
Wang and Zhang [71]	36	133.1–167.8	3.31–5.44	396–504	332-392	35.4–64.8	1140–2480
Zhao et al. [72]	9	90	1–1.5	300	329	29.6	342–391
Chang et al. [73]	3	111.64,113.64	1.9,3.64	400	261.3,259.6	47.8,56.7	666.6–1011
Guler et al. [74]	7	75.84,76.21	2.48–3.31	300	278–305	145	752–841
Guler et al. [75]	7	114.23–114.33	3.02–5.98	400	306–314	115	1216–1830
Ho and Lai [76]	5	114,168	5,8	330	365	29.1–114.3	1876–3101
Jin et al. [77]	3	198.9–200.3	1.99–2.98	398–401	402.5	54	2970–3230
Lee et al. [78]	2	300,360	6,12	900,1080	479,498	31.5	5550,6750
Lin [79]	6	150	0.7–2.1	80–800	247	23–36	513.52–1073.1
Tan [80]	16	108–133	1–7	378–465	232,429	77.4,84.7	1239–3404
Xiong et al. [81]	16	114.3,219.1	3.6-10	250,600	300,428	51.6–193.3	2314–9187
Luksha and Nesterovich [29]	10	159–1020	5.07–13.25	447–3060	312–381.5	15–41.5	2230–46,000
Sakino [30]	12	174–179	3–9	360	249–283	22.2–45.7	1220–2730
Total	478	60–1020	0.7–13.25	80–3060	185.7–835	15–193.3	312–46,000

**Table 2:** Optimal D/t ratios corresponding to different material strength matches.

		$f_{c}$ (MPa)	20	30	40	50	60	70	80	90	100
	D/t										
$f_{y}$ (MPa)											
235			51.98	52.48	52.73	51.84	50.65	48.80	46.34	43.64	40.74
345			69.42	68.87	68.25	66.55	64.57	62.43	59.33	56.25	53.16
390			76.56	75.56	74.56	72.40	70.24	67.88	64.81	61.90	58.24
420			81.40	79.83	78.68	76.17	73.85	71.61	68.40	65.00	61.25
500			94.08	91.18	89.35	86.50	83.94	80.74	77.27	73.53	70.00
600			109.51	105.12	102.31	98.57	95.17	91.74	87.89	84.00	80.00
700			124.05	118.16	114.29	110.00	105.77	101.90	98.13	93.85	89.00
800			137.58	130.29	125.63	120.71	116.52	111.58	107.33	102.50	97.00
900			149.68	141.56	136.21	130.77	126.19	121.18	116.43	110.91	105.00

## Data Availability

Some or all data will be available upon request from the corresponding author.
